# Comparison of Work-Related Stress in Cluster of Workers’ Suicides in Korea: Analysis of Industrial Accident Compensation Insurance, 2010–2017

**DOI:** 10.3390/ijerph19053013

**Published:** 2022-03-04

**Authors:** Jungwon Jang, Inah Kim, Yangwoo Kim, Jaechul Song

**Affiliations:** 1 Institute for Health and Society, Hanyang University, Seoul 04763, Korea; jwjang@hanyang.ac.kr; 2 Department of Occupational and Environmental Medicine, College of Medicine, Hanyang University, Seoul 04763, Korea; jsong@hanyang.ac.kr; 3 Department of Occupational and Environmental Medicine, Hanyang University, Seoul Hospital, Seoul 04763, Korea; oem.ywkim@gmail.com

**Keywords:** suicide, work-related stress, psychosocial factor, cluster analysis, responsibility, role change, physical risk, job insecurity, interpersonal conflict, industrial accident compensation insurance

## Abstract

Background: There is limited research on the heterogeneity of worker suicides. We compared differences in workers’ suicides by clustering suicide deaths. Methods: From 2010 to 2017, 353 suicide deaths were claimed in the Industrial Accident Compensation Insurance; variables were coded using a standardized methodology. A two-step cluster analysis classified the clusters based on demographic and employment conditions. Details of the suicide, clinical variables, personal stresses, and work-related stresses were compared using the chi-square test and one-way analysis of variance. Results: We identified five clusters and they differed particularly in work-related stress. “Responsibility-burdened type” experienced excessive responsibility as managers; “role-changed type” experienced a sudden and unpredictable role change as clerks or sales workers; “risk-exposed type” experienced physical risk factors at work (working alone, outdoors, and in shifts) as machine operating and assembling workers, or craft and related trades workers; “job-insecurity type” experienced unstable employment (irregular, nonpermanent) as elementary or service workers; “workplace-violence type” was mainly unmarried women who lived alone, and experienced interpersonal conflict and violence as professionals and related workers. There were no differences between clusters in clinical variables (except problem drinking) and personal stresses. Conclusion: Interventions to alleviate work-related stress in worker clusters are needed to prevent suicide in workers.

## 1. Introduction

Suicide is one of the leading causes of death among the working population in Korea, taking first place among those aged 10–39 years, and second for 40–59-year-olds [1]. In 2017, approximately 9000 people of the Korean working population (aged 15–64 years) died by suicide. This indicates an increase of about 57% (from 15.5 per 100,000 population in 2000 to 24.3 in 2017) in less than 20 years.

Recently, suicide studies have been conducted to classify clusters of suicide deaths in the general population [2,3,4,5]. As individual experiences and conditions that influence suicide are very diverse, it is necessary to classify suicide deaths into subgroups according to similarities and differences [3].

Work-related stress is an important factor in the study of worker suicide because long-term exposure to negative psychosocial factors at work is related to mental health problems, such as depression, which increase the risk of suicide [6,7]. Studies on work-related stress and suicide death among workers are relatively rare. Factors such as chronobiological and physical working conditions [8], low occupational grade [9], work injury, job insecurity, conflict with supervisors/colleagues [10], low job control [11], and low psychological job demand [12] may lead to work-related stress and were associated with suicidal death. However, these studies were conducted without classifying suicidal deaths into subgroups. As work-related stress can vary from worker to worker as it arises from negative interactions with workers’ personal experiences and work conditions [13], it is necessary to classify subgroups and compare characteristics that affect suicide, for effective prevention of workers’ suicide [2,4].

To the best of our knowledge, no attempt has been made to cluster workers based on demographic and employment conditions suicide and compare its work-related stress. We aimed to classify workers’ suicide deaths into subgroups and compare different patterns of work-related stresses, using data from all claimed suicide deaths in Industrial Accident Compensation Insurance (IACI) from 2010 to 2017 in Korea.

## 2. Materials and Methods

### 2.1. Industrial Accident Compensation Insurance Data

When the bereaved family of a worker submits the application form for compensation to the Workers’ Compensation & Welfare Service of Korea (COMWEL, Ulsan, Korea) to obtain approval for work-related stress as cause of suicide, COMWEL identifies suicide using death certification, police report, etc. COMWEL thoroughly collects all available information and conducts investigations to examine work-related stresses, personal stresses, and demographics six months before the suicide. IACI data on each claim contain an investigation report by COMWEL that includes all relevant evidence: death certification, police report, National Health Insurance Service care benefit statement, medical records, evidence of work (such as employment contract and job assignment), interviews or letters (from employers, colleagues, family, friends, witnesses, etc.), suicide notes, diary entries, emails, and text messages. The Committee on Occupational Disease Judgement (Korea) advises on the investigation plans and prepares a Decision Statement after judging the relevance of work-related stress based on the investigation report.

This study used data from all 353 suicide death claims to the IACI from 2010 to 2017 that were determined as suicide by COMWEL. COMWEL allowed researchers to use IACI data in accordance with ethical principles, and provided it in text format with all personal information deleted. All procedures were carried out with the approval of the Institutional Review Board at Hanyang University (Seoul, Korea).

### 2.2. Variable Coding

To extract variables from IACI data, coding schemes were constructed by referring to previous suicide studies [4,14,15]. All variables were numerically coded as either categorical or continuous. When it was difficult to judge work-related stress, it was coded as missing data. Two authors independently coded the same 30 cases for inter-rater agreement. According to Landis and Koch’s (1997) [16] standard, kappa values of demographics, details of the suicide, clinical variables, and employment conditions (0.87–1.00) were almost perfect, and those of personal stresses and work-related stresses (0.65–0.92) were substantial or almost perfect. However, those of variety of work, interestingness of work, satisfaction with supervisor, and satisfaction with colleagues were low (0.20–0.33), so they were excluded from the final variables. The first author coded all the other cases for consistency and another author reviewed the codes. The corresponding author supervised the entire coding process. A more detailed coding method has been described in our previous study [17].

The final coding schemes and variables that were extracted comprised: (1) demographics: age at death, sex, marital status, living conditions; (2) details of the suicide: method of suicide, location of suicide, suicide note; (3) clinical variables: history of psychiatric treatment, past diagnosis of mental disorders, previous suicide attempts, family history of mental disorders/suicide/suicide attempts, contact with psychiatry and/or emergency department services within the last one month, history of physical illness, drinking, problem drinking, smoking; (4) employment conditions: years of continuous employment, occupation, employment contract, status of workers, employment status at the time of death, main place of work, shift work, emotional labor; (5) personal stresses: family problems, illness in family, death of a family member/relative/friend, financial problems, interpersonal conflict with close relationships; (6) work-related stresses: 36 previously described [17] work-related stress variables from the 2011 Recognition Criteria for Occupation Mental Disorders in Japan [18], and work-related stress as listed in the Occupational Stress Event Checklist in COMWEL [19]: (i) Accident/Disaster: traffic accidents and poor physical work environment, (ii) Failure/Responsibility: no variables to add, (iii) Quantity/Quality: high levels of time pressure and lack of control over work, (iv) Role/Position: a sudden change in work-related matters, mismatch between work and ability/skill, and changing jobs, and (v) Interpersonal Conflict: no variables to add.

### 2.3. Statistical Analysis

A two-step cluster analysis method was used to classify the clusters of worker suicide deaths. Cluster analysis is a statistical technique that meaningfully combines several homogeneous clusters based on similarities and differences between observed individuals (or objects), and has been used as a basis for the cause-identification and treatment of suicide risk [20]. Two-step cluster analysis has the advantage of processing categorical and continuous variables at the same time and automatically selecting the number of clusters based on statistical evaluation criteria [21].

We established three criteria when selecting clustering variables. First, as work-related stress arises from negative interactions with workers’ personal experiences and work conditions [13], the variables of demographic and employment conditions were included. Second, as the purpose of classifying suicide deaths into subgroups is to compare work-related stresses that can influence suicide-related behavior [4], variables known to directly influence suicide behavior, such as mental disorders, past suicide attempts, and problem drinking [22,23], were excluded from clustering. Third, clustering variables were selected from among the variables without missing values.

Final clustering variables included demographic (age at death, sex, marital status, living conditions) and employment conditions (occupation, employment contract, status of workers, employment status at the time of death, main place of work). Age at death was a continuous variable while others were categorical. Log-likelihood was used to measure the cluster distance. The optimal number of clusters was determined through a statistical program according to Schwartz’s Bayesian Information criterion. The silhouette measures of cohesion and segregation indicating the goodness-of-fit of the cluster were identified. A generally accepted criterion is that the silhouette measure of <0.2 is considered lack, between 0.2–0.5 indicates a fair solution, and >0.5 is a reasonable classification [20,21]. After classifying the clusters, the chi-square test (or Fisher’s exact test) for categorical variables, and one-way analysis of variance for continuous variables were conducted to compare differences in clusters. The statistical significance level was 0.05. All analyses were performed using IBM SPSS Statistics 26.0 (IBM Corp., Armonk, NY., USA).

## 3. Results

From the cluster analysis of 353 suicide deaths, five distinct clusters were determined, with an average silhouette measure of 0.3. This silhouette measure was a fair solution and was identical to that of previous suicide cluster studies [4,5]. The clustering variables from most to least important were employment contract, marital status, occupation, main place of work, status of workers, age at death, sex, living conditions, and employment status at the time of death.

Table 1 shows the comparison results by cluster for demographic, suicide details, and clinical variables. Except for problem drinking among the clinical variables, there were no significant differences in details of the suicide and clinical variables. A comparison of employment conditions by cluster is shown in Table 2. Table 3 shows the comparison of personal stresses and work-related stresses by cluster. Only work-related stresses with significant differences are presented in Table 3.

Cluster 1 was the largest cluster (*n* = 108). All workers were married men who did not live alone (100%). Of those, most were in their 40s (51.9%) and worked as managers (61.1%). Workers in this cluster had the longest duration of continuous employment (mean = 13.9 years). This cluster was mainly related to excessive responsibility, with a higher proportion than other clusters. In the Fail/Responsibility variable, work-related stresses included “difficult work to achieve” (49.1%), “fail to achieve allocation workload” (38.9%), and “in charge of new business or company reconstruction” (22.2%); the Quantity/Quality variable included “change of job contents or workload” (44.4%) and “high levels of time pressure” (44.4%); the Role/Position variable included “own promotion” (21.3%); in the Interpersonal Conflict variable, work-related stresses included “conflict with supervisor” (44.4%).

Cluster 2 (*n* = 71) included clerks (49.3%) or sales workers (16.9%). Work-related stress was mainly related to a change of role: “a sudden change in work-related matters” (42.9%), “personnel changes” (38.0%), and “mismatch between work and ability/skill” (30.6%) in Role/Position; and “change of pace or activity” (31.0%) and “change in work arrangements of shift” (23.9%) in Quantity/Quality.

Cluster 3 (*n* = 74) included the oldest (mean = 47.6 years) and the highest proportion of divorced/widowed workers (14.9%). This cluster included workers who worked outdoors (including transportation; 71.6%) and did shift work (23.0%) as equipment, machine operating, and assembling workers (28.4%), or as craft and related trades workers (20.3%). Their work-related stress was attributable to physical risk factors: “lack of control over work (workload, work pace)” (62.2%) in Quality/Quantity; “poor physical work environment” (41.9%) and “traffic accidents” (13.5%) in Accident/Disaster; and “working alone” (39.4%) in Role/Position.

Cluster 4 was the smallest cluster (*n* = 44). It included the highest proportion of workers over 60 years old and workers who had problem drinking behaviors (both 18.2%), compared with other clusters. Cluster 4 included irregular (100%), nonpermanent (50.0%), unemployed at the time of death (29.5%) as elementary (36.4%), or service workers (11.4%). Workers in this cluster had the shortest duration of continuous employment (mean = 2.8 years). Work-related stress was associated with unstable employment: “expiration of contract” (18.6%), “changing jobs” (16.3%), and “discrimination (due to irregular worker)” (9.3%) in Role/Position; “severe disease/injury” (18.6%) in Accident/Disaster; and “forcing illegal behavior” (4.7%) in Fail/Responsibility.

Cluster 5 (*n* = 56) included the youngest (mean = 31.3 years; 83.9% were less than 40 years) workers. All were unmarried (single or divorced/widowed). Workers in this cluster were women (33.9%), those who lived alone (28.6%), and professionals and related workers (35.7%). Work-related stresses were mostly attributed to workplace violence: “conflict with colleague” (35.3%), “workplace harassment, mobbing, violence” (16.7%), and “sexual harassment” (3.6%) in Interpersonal Conflict.

## 4. Discussion

Our study classified 353 workers, who died by suicide, across five subgroups. All five clusters showed different patterns, especially in work-related stress. The identified clusters were named according to the patterns of work-related stress as follows: Responsibility-burdened type, Role-changed type, Risk-exposed type, Job insecurity type, and Workplace-violence type.

### 4.1. Cluster 1 (Responsibility-Burdened Type)

Cluster 1 (Responsibility-burdened type) included all married men, mostly managers in their 40 s, who mainly experienced excessive responsibility associated with “difficult work to achieve,” “fail to achieve allocation workload,” “change of job contents or workload,” “high levels of time pressure,” “in charge of new business or company reconstruction,” and “own promotion.” This cluster represents a recently increasing type of suicide in Korea. As noted earlier [17,24], the reason the suicide rate of white-collar workers such as managers is increasing in Korea is that they are overworked in the face of tremendous competition and experience performance pressures. Similarly, in Japan, mental disorders and suicides caused by overwork are rapidly increasing [25], and people in management positions are dying by suicide to take responsibility for serious work-related stressful events [26]. The social culture of Korea and Japan, which places excessive importance on responsibility for one’s work, seems to have a negative effect on the mental health of workers. In general, the low probability of promotion was reported to be related to workers’ mental health problems [27]. However, an analysis of reports related to compensation insurance for 22 workers who died by suicide in Japan confirmed that promotion also played a role in suicide [14]. The experience of promotion in Cluster 1 is assumed to be linked to an increased burden of responsibility and overwork.

To prevent suicide among workers such as in Cluster 1, it is necessary to deal with the problem of overwork. Raising public awareness of the effects of overwork on workers’ mental health and changing organizational culture can be effective in preventing occupational mental disorders and suicide [24].

### 4.2. Cluster 2 (Role-Changed Type)

Cluster 2 (Role-changed type) comprised clerks or sales workers who experienced work-related stress attributable to a change of role, such as “a sudden change in work-related matters,” “personnel changes,” “mismatch between work and ability/skill,” “change of pace or activity,” and “change in work arrangements of shift.” Workers who have undergone undesirable or drastic changes in the workplace undergo mental suffering. A prospective cohort study in France found that major changes in work content or in the organization were predictive of depressive symptoms [6]. In particular, role conflict caused by the experience of unpredictable or unwanted role changes increased workers’ emotional distress [28,29]. Japanese studies reported a high percentage of those who experienced unwanted transfers in the workplace among those who died by or attempted suicide [14,30]. These results suggest the importance of communication about changes in organizational culture.

When planning organizational or role changes, senior management should discuss major changes with the concerned workers in advance, to enable them to cope with stressful changes [28,29]. They should also provide relevant support for mental health.

### 4.3. Cluster 3 (Risk-Exposed Type)

Cluster 3 (Risk-exposed type) included workers who were the oldest, were divorced/widowed, and were mainly equipment, machine operating, and assembling workers, or craft and related trades workers. Most of them worked outdoors (including transportation) and did shift work. Their work-related stress was attributed to physical risk factors such as “lack of control over work (workload, work pace)”, “working alone”, “poor physical work environment”, and “traffic accidents”. A longitudinal study in Germany found that a high total score of chronobiological (e.g., shift work, night shift, or assembly-line work) and physical factors (e.g., hazardous work, noise, or pollutant) increased the risk of suicide by about three times [8]. This finding supports Cluster 3 that included workers who worked in shifts and in hazardous physical environments. Shift work causes sleep disturbances, which can increase the risk of suicide [31,32]. The finding that Cluster 3 had the highest rate of “lack of control over work” may also be attributable to type of work, such as shift work.

Night-shift work increases the risk of traffic accidents [33]. Subway drivers who encountered a person-under-train experienced a fourfold increase in panic disorder and post-traumatic stress disorder [34]. Individuals with these disorders are at a higher risk of suicide [35]. Although not statistically significant, Cluster 3 had the highest rate of anxiety disorders among principal diagnoses of mental disorders.

Therefore, to prevent suicide among the type of workers in this cluster, it is necessary to consider an optimization of shift-work schedules that does not disturb the circadian rhythm and does not cause sleep disturbance for occupations where shift work is inevitable [36]. In addition, safety management at the workplace is essential to prevent casualties. After an accident, active intervention in the workplace is required to treat workers’ trauma [34].

### 4.4. Cluster 4 (Job-Insecurity Type)

Cluster 4 (Job-insecurity type) included elementary or service workers. They worked as irregular and nonpermanent workers and had the shortest period of continuous employment. The proportion of unemployment at the time of death was highest compared to other clusters. Cluster 4 comprised those “between employment and unemployment” [37], as work-related stress was attributed to “expiration of contract,” “changing jobs,” and “discrimination (due to irregular worker),” which are associated with job insecurity. Form of job insecurity, such as unemployment and temporary or fixed-term contracts, is known to be the major factor that increases the risk of suicide [15]. In Cluster 4, unemployment at the time of death is estimated to be more likely to be involuntary, due to contract termination. Involuntary unemployment increases the risk of suicide [38]. Moreover, the highest proportion of problem drinking behavior observed in Cluster 4 may also be associated with job insecurity. Job insecurity is associated with increased alcohol use [39], and problem drinking behaviors significantly increase the risk of suicide death [22].

Suicide prevention for workers suffering from job instability requires active intervention and efforts of the state and society to lower the unemployment rate and eliminate irregular workers. The “active labor market program” in Europe is known as an effective way to reduce the suicide rate by alleviating health side-effects caused by unemployment [40].

### 4.5. Cluster 5 (Workplace-Violence Type)

Cluster 5 (Workplace-violence type) included the youngest workers, the unmarried (single or divorced/widowed), and the highest proportion of female workers living alone. They worked primarily as professionals and related workers. They experienced the most interpersonal conflict and violence such as “conflict with colleague”, “workplace harassment, mobbing, violence”, and “sexual harassment”. Violence in the workplace is significantly associated with increased suicidal ideation [41]. Lack of support and interaction with superiors and colleagues may also increase the risk of suicide [42], whereas social support is effective in preventing suicide [43]. As Cluster 5 included the youngest workers, they may have experienced problems adjusting to the workplace as new employees. New employees who need to adapt to social groups may be more vulnerable to workplace violence, and may require more social support. However, Cluster 5 workers may have lacked protective factors, such as social support, due to living alone [43].

In a Japanese study, women reported about twice as much workplace bullying as men [44]. In IACI-approved cases of mental health, women had higher interpersonal conflicts [45]. The results of Cluster 5 may be characteristic of professionals and related workers (e.g., nurses), which mainly comprise women. The Japanese study cannot be compared with Cluster 5, due to the lack of occupational information, so further research on occupation and gender is needed.

As workplace bullying has emerged as a serious social issue in Korea, the Labor Standards Act was amended in July 2019 to prohibit workplace bullying and to stipulate the obligation to take action in cases of bullying. Every workplace should make efforts to prevent workplace harassment by preparing countermeasures and providing interventions for workers to adapt to their workplace [41].

## 5. Strengths and Limitations

To the best of our knowledge, this is the first study to classify clusters of worker suicide deaths by demographic and employment conditions and to compare details of the suicide, clinical variables, personal stresses, and work-related stresses into clusters, using data on suicide deaths of all workers who claimed IACI over eight years in South Korea. In particular, the difference in work-related stress by cluster confirmed the need for a detailed work-related stress intervention strategy to prevent worker suicide.

Notably, there were no differences between clusters in clinical variables (except problem drinking) and personal stresses known as suicide risk factors in previous studies, such as mental disorders, past suicide attempts, family history of mental disorders or suicide/suicide attempts, physical illness, family problems, financial problems, and interpersonal conflict in close relationships [7,23]. While workers who died by suicide experienced similar levels of clinical variables and personal stress, it is possible to speculate that work-related stress may have played a role in triggering suicide. However, further research is needed to clarify this.

This study had certain limitations. First, the data of this study were not completely representative of the suicide deaths of Korean workers, because it only included those who applied for IACI. The economically inactive population, unemployed persons, workers who are compensated by other laws, businesses with fewer than five workers, and private households with employed persons are not covered under the IACI. The IACI only includes data of workers whose bereaved family claimed insurance after their suicide, and does not include data where no compensation was claimed. Second, there is the possibility of information or recall bias in the contents of IACI data. Whereas information confirmed through the death certification, police report, National Health Insurance Service care benefit statement, medical records, and evidence of work is highly accurate, personal and work-related stress identified through interviews/letters from employers, colleagues, family, close friends, or related persons may be somewhat less valid. However, researchers understand that the validity of the IACI data is higher than that of a psychological autopsy, which is primarily informed by interviews, because COMWEL confirms the facts of the interviews/letters through all relevant evidence. Third, there is the possibility of errors in cluster classification. The silhouette value indicating the quality of the cluster in this study is 0.3, which is a fair solution [20], and the same as those of the previous suicide cluster studies [4,5]. However, the clusters seem to overlap because of the high proportion of married male regular workers. Due to the limitation of the variables that can be obtained from IACI data, only demographic and employment conditions were used for clustering variables. Further research is necessary to classify subgroups using various factors such as social or external environment that can affect workers’ suicide.

## 6. Conclusions

Our findings suggest that work-related stress may work in conjunction with demographic and employment conditions to have an impact on suicide among workers. As these factors may vary across subgroups it is necessary to comprehensively consider various characteristics of workers such as demographic and employment conditions when intervening to prevent worker suicide and promote mental health. Suicide prevention programs and policies should be applied to alleviate the work-related stress of each subgroup to effectively reduce suicide among workers.

## Figures and Tables

**Table 1:** Comparison of demographic, suicide details, and clinical variables for five clusters of worker suicide deaths.

Variables	Cluster 1 (*n* = 108)		Cluster 2 (*n* = 71)		Cluster 3 (*n* = 74)		Cluster 4 (*n* = 44)		Cluster = 5 (*n* = 56)		Total (*n* = 353)		*p*-Value
Demographics, *n* (%)													
Age at death, year, mean (SD)	45.2	(6.7)	43.1	(6.4)	47.6	(8.9)	46.7	(13.6)	31.3	(6.6)	43.3	(9.8)	<0.0001
Age at death groups, year													<0.0001
<30	0	(0)	0	(0)	0	(0)	6	(13.6)	27	(48.2)	33	(9.3)	
30–39	20	(18.5)	25	(35.2)	14	(18.9)	7	(15.9)	20	(35.7)	86	(24.4)	
40–49	56	(51.9)	31	(43.7)	32	(43.2)	11	(25.0)	8	(14.3)	138	(39.1)	
50–59	31	(28.7)	15	(21.1)	22	(29.7)	12	(27.3)	1	(1.8)	81	(22.9)	
≥60	1	(0.9)	0	(0)	6	(8.1)	8	(18.2)	0	(0)	15	(4.2)	
Sex													<0.0001
men	108	(100)	61	(85.9)	73	(98.6)	38	(86.4)	37	(66.1)	317	(89.8)	
women	0	(0)	10	(14.1)	1	(1.4)	6	(13.6)	19	(33.9)	36	(10.2)	
Marital status													< 0.0001
single (never married)	0	(0)	0	(0)	0	(0)	9	(20.5)	53	(94.6)	62	(17.6)	
married	108	(100)	71	(100)	63	(85.1)	30	(68.2)	0	(0)	272	(77.1)	
divorced/widowed	0	(0)	0	(0)	11	(14.9)	5	(11.4)	3	(5.4)	19	(5.4)	
Living conditions													<0.0001
living alone	0	(0)	2	(2.8)	9	(12.2)	4	(9.1)	16	(28.6)	31	(8.8)	
not living alone	108	(100)	69	(97.2)	65	(87.8)	40	(90.9)	40	(71.4)	322	(91.2)	
Details of the suicide, *n* (%)													
Method of suicide													0.213
hanging	62	(57.4)	40	(57.1)	43	(58.1)	14	(32.6)	29	(53.7)	188	(53.9)	
jumping from height	28	(25.9)	17	(24.3)	15	(20.3)	18	(41.9)	14	(25.9)	92	(26.4)	
other	18	(16.7)	13	(18.6)	16	(21.6)	11	(25.6)	11	(20.4)	69	(19.8)	
Location of suicide													0.232
own residence	48	(44.4)	33	(46.5)	32	(43.2)	15	(34.9)	29	(52.7)	157	(44.7)	
workplace	23	(21.3)	19	(26.8)	13	(17.6)	15	(34.9)	7	(12.7)	77	(21.9)	
other	37	(34.3)	19	(26.8)	29	(39.2)	13	(30.2)	19	(34.5)	117	(33.3)	
Suicide note, yes	36	(33.3)	25	(36.2)	27	(36.5)	15	(35.7)	17	(32.1)	120	(34.7)	0.979
Clinical variables, *n* (%)													
History of psychiatric treatment, yes	57	(53.3)	40	(56.3)	43	(58.1)	20	(46.5)	29	(52.7)	189	(54.0)	0.793
Past diagnosis of mental disorders													0.180
depressive disorders	29	(50.0)	25	(62.5)	20	(46.5)	11	(55.0)	15	(53.6)	100	(52.9)	
anxiety disorders	21	(36.2)	11	(27.5)	17	(39.5)	5	(25.0)	4	(14.3)	58	(30.7)	
others	8	(13.8)	4	(10.0)	6	(14.0)	4	(20.0)	9	(32.1)	31	(16.4)	
Previous suicide attempts, yes	9	(8.3)	8	(11.8)	7	(9.6)	6	(15.8)	7	(13.5)	37	(10.9)	0.699
Family history of mental disorders, yes	4	(4.4)	7	(12.5)	2	(3.3)	4	(16.7)	4	(9.8)	21	(7.7)	0.081
Family history of suicide/suicide attempts, yes	1	(1.1)	3	(5.4)	1	(1.6)	0	(0)	0	(0)	5	(1.8)	0.381
Contact with psychiatry and/or emergency department services within the last 1 month, yes	54	(55.1)	33	(58.9)	40	(58.8)	22	(57.9)	28	(60.9)	177	(57.8)	0.971
History of physical illness, yes	58	(53.7)	36	(52.2)	45	(60.8)	21	(53.8)	22	(40.0)	182	(52.8)	0.232
Drinking, yes	78	(76.5)	50	(72.5)	61	(85.9)	27	(71.1)	38	(79.2)	254	(77.4)	0.295
Problem drinking, yes	3	(2.8)	2	(2.8)	10	(13.5)	8	(18.2)	5	(9.1)	28	(8.0)	0.003
Smoking, yes	53	(52.0)	31	(47.0)	37	(54.4)	20	(55.6)	22	(45.8)	163	(50.9)	0.810

**Table 2:** Comparison of employment conditions for five clusters of worker suicide deaths.

Employment Conditions	Cluster 1 (*n* = 108)		Cluster 2 (*n* = 71)		Cluster 3 (*n* = 74)		Cluster 4 (*n* = 44)		Cluster = 5 (*n* = 56)		Total (*n* = 353)		*p*-Value
Years of continuous employment, year, mean (SD)	13.9	(9.2)	13.6	(7.4)	10.9	(9.4)	2.8	(3.5)	4.9	(6.2)	10.4	(9.0)	<0.0001
Occupation, *n* (%)													<0.0001
managers	66	(61.1)	1	(1.4)	17	(23.0)	7	(15.9)	6	(10.7)	97	(27.5)	
professionals and related workers	25	(23.1)	0	(0)	4	(5.4)	4	(9.1)	20	(35.7)	53	(15.0)	
clerks	0	(0)	35	(49.3)	0	(0)	1	(2.3)	11	(19.6)	47	(13.3)	
service workers	0	(0)	3	(4.2)	2	(2.7)	5	(11.4)	1	(1.8)	11	(3.1)	
sales workers	0	(0)	12	(16.9)	4	(5.4)	2	(4.5)	3	(5.4)	21	(5.9)	
skilled agricultural, forestry, and fishery workers	0	(0)	0	(0)	1	(1.4)	0	(0)	0	(0)	1	(0.3)	
craft and related trades workers	17	(15.7)	0	(0)	15	(20.3)	6	(13.6)	6	(10.7)	44	(12.5)	
equipment, machine operating and assembling workers	0	(0)	20	(28.2)	21	(28.4)	3	(6.8)	4	(7.1)	48	(13.6)	
elementary workers	0	(0)	0	(0)	10	(13.5)	16	(36.4)	5	(8.9)	31	(8.8)	
Employment contract, *n* (%)													<0.0001
regular	108	(100)	71	(100)	74	(100)	0	(0)	54	(96.4)	307	(87.0)	
irregular	0	(0)	0	(0)	0	(0)	44	(100)	2	(3.6)	46	(13.0)	
Status of workers, *n* (%)													<0.0001
permanent	108	(100)	71	(100)	74	(100)	22	(50.0)	56	(100)	331	(93.8)	
nonpermanent	0	(0)	0	(0)	0	(0)	22	(50.0)	0	(0)	22	(6.2)	
Employment status at the time of death, *n* (%)													<0.0001
employed	108	(100)	67	(94.4)	57	(77.0)	31	(70.5)	51	(91.1)	314	(89.0)	
unemployed	0	(0)	4	(5.6)	17	(23.0)	13	(29.5)	5	(8.9)	39	(11.0)	
Main place of work, *n* (%)													<0.0001
indoors	108	(100)	71	(100)	21	(28.4)	28	(63.6)	51	(91.1)	279	(79.0)	
outdoors (including transportation)	0	(0)	0	(0)	53	(71.6)	16	(36.4)	5	(8.9)	74	(21.0)	
Shift work, yes	5	(4.6)	9	(12.7)	17	(23.0)	7	(17.1)	9	(16.4)	47	(13.5)	0.008
Emotional labor, yes	26	(24.1)	25	(35.2)	25	(34.2)	15	(35.7)	18	(32.7)	109	(31.2)	0.425

**Table 3:** Comparison of personal stresses and work-related stresses for five clusters of worker suicide deaths.

Stresses	Cluster 1 (*n* = 108)		Cluster 2 (*n* = 71)		Cluster 3 (*n* = 74)		Cluster 4 (*n* = 44)		Cluster 5 (*n* = 56)		Total (*n* = 353)		*p*-Value
Personal Stresses, yes, *n* (%)													
Family problems	7	(6.5)	6	(8.6)	8	(11.3)	7	(17.5)	5	(9.8)	33	(9.7)	0.369
Illness in family	10	(9.4)	2	(2.9)	3	(4.1)	0	(0)	6	(12.2)	21	(6.3)	0.065
Death of a family member/relative/friend	5	(4.6)	7	(10.0)	5	(6.8)	1	(2.5)	2	(3.7)	20	(5.8)	0.490
Financial problems	11	(10.6)	10	(14.7)	18	(25.4)	7	(18.9)	7	(14.9)	53	(16.2)	0.127
Interpersonal conflict with close relationships	4	(3.7)	5	(7.4)	4	(5.7)	7	(19.4)	4	(8.0)	24	(7.3)	0.058
Work-related stresses, yes, *n* (%) *													
Accident/Disaster													
Severe disease/injury	7	(6.5)	6	(8.5)	12	(16.2)	8	(18.6)	3	(5.4)	36	(10.2)	0.049
Traffic accidents	2	(1.9)	3	(4.2)	10	(13.5)	1	(2.3)	0	(0)	16	(4.6)	0.002
Poor physical work environment	9	(8.4)	6	(8.5)	31	(41.9)	13	(30.2)	5	(9.1)	64	(18.3)	<0.0001
Failure/Responsibility													
Forcing illegal behavior	0	(0)	3	(4.2)	0	(0)	2	(4.7)	1	(1.8)	6	(1.7)	0.030
Difficult work to achieve	53	(49.1)	28	(39.4)	21	(28.8)	10	(23.3)	15	(27.3)	127	(36.3)	0.005
Fail to achieve allocation workload	42	(38.9)	20	(28.2)	12	(16.4)	5	(11.6)	10	(18.2)	89	(25.4)	0.001
In charge of new business or company reconstruction	24	(22.2)	9	(12.7)	9	(12.2)	1	(2.3)	3	(5.4)	46	(13.1)	0.004
Quantity/Quality													
Change of job contents or workload	48	(44.4)	29	(40.8)	11	(14.9)	6	(14.0)	19	(33.9)	113	(32.1)	<0.0001
Change in work arrangements of shift	19	(17.6)	17	(23.9)	2	(2.7)	2	(4.7)	3	(5.4)	43	(12.3)	<0.0001
Change of pace or activity	29	(26.9)	22	(31.0)	5	(6.8)	3	(7.0)	5	(8.9)	64	(18.2)	<0.0001
High levels of time pressure	48	(44.4)	16	(22.5)	30	(41.1)	6	(14.3)	12	(21.8)	112	(32.1)	<0.0001
Lack of control over work (workload, work pace)	31	(29.0)	39	(54.9)	46	(62.2)	24	(57.1)	30	(54.5)	170	(48.7)	<0.0001
Role/Position													
Personnel changes	29	(27.1)	27	(38.0)	9	(12.2)	5	(11.6)	6	(10.9)	76	(21.7)	<0.0001
Working alone	8	(7.4)	8	(11.4)	28	(39.4)	5	(12.5)	6	(10.9)	55	(16.0)	<0.0001
Discrimination (due to irregular worker)	0	(0)	0	(0)	0	(0)	4	(9.3)	1	(1.8)	5	(1.4)	<0.0001
Own promotion	23	(21.3)	7	(9.9)	5	(6.8)	0	(0)	2	(3.6)	37	(10.5)	<0.0001
Expiration of contract	0	(0)	0	(0)	0	(0)	8	(18.6)	1	(1.8)	9	(2.6)	<0.0001
A sudden change in work-related matters	38	(38.8)	27	(42.9)	14	(20.3)	3	(8.3)	11	(21.2)	93	(29.2)	<0.0001
Mismatch between work and ability/skill	25	(26.6)	19	(30.6)	5	(7.4)	4	(11.1)	10	(24.4)	63	(20.9)	0.004
Changing jobs	4	(3.7)	0	(0)	4	(5.4)	7	(16.3)	3	(5.5)	18	(5.1)	0.005
Interpersonal Conflict													
Workplace harassment, mobbing, violence	6	(5.6)	3	(4.2)	1	(1.4)	1	(2.4)	9	(16.7)	20	(5.7)	0.010
Conflict with supervisor	48	(44.4)	28	(40.0)	16	(22.5)	10	(27.0)	22	(40.7)	124	(36.5)	0.025
Conflict with colleague	13	(12.3)	10	(14.3)	8	(10.8)	8	(22.2)	18	(35.3)	57	(16.9)	0.002
Sexual harassment	0	(0)	0	(0)	0	(0)	0	(0)	2	(3.6)	2	(0.6)	0.038

* Only work-related stresses with significant differences by cluster are presented in Table 3.

## Data Availability

The datasets generated during the current study are available from the corresponding author on reasonable request.
